# How should we interpret lactate in labour? A reference study

**DOI:** 10.1111/1471-0528.17264

**Published:** 2022-08-08

**Authors:** Samuel Dockree, Joseph O'Sullivan, Brian Shine, Tim James, Manu Vatish

**Affiliations:** ^1^ Women's Centre John Radcliffe Hospital Oxford University Hospitals NHS Foundation Trust Oxford UK; ^2^ Department of Clinical Biochemistry John Radcliffe Hospital Oxford University Hospitals NHS Foundation Trust Oxford UK; ^3^ Women's Centre Nuffield Department of Women's and Reproductive Health John Radcliffe Hospital University of Oxford Oxford UK

**Keywords:** infection, labour, lactate, perinatal, puerperium, sepsis

## Abstract

**Objective:**

To investigate maternal lactate concentrations in labour and the puerperium.

**Design:**

Reference study.

**Setting:**

Tertiary obstetric unit.

**Population:**

1279 pregnant women with good perinatal outcomes at term.

**Methods:**

Electronic patient records were searched for women who had lactate measured on the day of delivery or in the following 24 hours, but who were subsequently found to have a very low likelihood of sepsis, based on their outcomes.

**Main outcome measures:**

The normative distribution of lactate and C‐reactive protein (CRP), differences according to the mode of birth, and the proportion of results above the commonly used cut‐offs (≥2 and ≥4 mmol/l).

**Results:**

Lactate varied between 0.4–5.4 mmol/l (median 1.8 mmol/l, interquartile range [IQR] 1.3–2.5). It was higher in women who had vaginal deliveries than caesarean sections (median 1.9 vs. 1.6 mmol/l, *p*_diff_ < 0.001), demonstrating the association with labour (particularly active pushing in the second stage). In contrast, CRP was more elevated in women who had caesarean sections (median 71.8 mg/l) than those who had vaginal deliveries (33.4 mg/l, *p*_diff_ < 0.001). In total, 40.8% had a lactate ≥2 mmol/l, but 95.3% were <4 mmol/l.

**Conclusions:**

Lactate in labour and the puerperium is commonly elevated above the levels expected in healthy pregnant or non‐pregnant women. There is a paucity of evidence to support using lactate or CRP to make decisions about antibiotics around the time of delivery but, as lactate is rarely higher than 4 mmol/l, this upper limit may still represent a useful severity marker for the investigation and management of sepsis in labour.

## 1 INTRODUCTION

Sepsis remains the third most common cause of maternal death worldwide.^1^ This is also true in the UK: 21 women died of sepsis between 2017 and 2019, constituting a maternal mortality rate of 1.1 per 100 000 pregnancies.^2^ There are ongoing initiatives to provide robust, evidence‐based guidance to improve the early recognition of maternal sepsis, notably including the importance of interpreting observations against pregnancy‐specific standards^3^, ^4^ and using diagnostic tools for objective clinical decision making.^5^, ^6^

Making an accurate diagnosis is essential for the timely administration of intravenous antibiotics and other interventions, which can be improved with pregnancy‐specific reference intervals.^7^ Unfortunately, unlike some specialities,^8^, ^9^ new and highly specific infection markers, such as procalcitonin, are seldom used in obstetrics, despite evidence supporting their validity in pregnancy.^10^, ^11^ This prompts us to look instead at the current most commonly used tests, to investigate how we can optimise them.

C‐reactive protein (CRP) and white blood cells (WBC) are commonly measured in pregnant women with suspected sepsis.^12^, ^13^ Although both markers have established (antenatal) pregnancy‐specific reference intervals, there is increasingly strong evidence they have limited value in labour and the postnatal period.^14^, ^15^, ^16^ Unlike CRP and WBC, which are usually measured to investigate the likelihood of disease and to inform decisions about antibiotics, lactate is a useful marker of severity in the ongoing management of sepsis,^17^ often requiring repeated measurements to investigate the trajectory.^18^, ^19^

In labour (a period of intense physical exertion and anaerobic respiration), lactate concentrations are expected to increase. Furthermore, dynamic changes in the maternal plasma volume will affect lactate, driven by dehydration, haemorrhage and the natriuretic effects of oxytocin. A recent review reported that lactate levels above 2 mmol/l were likely to be abnormal in pregnant women, as in the non‐pregnant population.^20^ However, lactate levels in labour were highly variable, and were frequently elevated above 4 mmol/l (which would otherwise usually denote end‐organ damage). If lactate is raised in normal labour and the postnatal period, it remains unclear how it should be interpreted to inform safe clinical decision making about the severity of sepsis in this high‐risk population.

To address this, we performed a large population study of pregnant women to investigate the distribution of lactate levels in women where the clinical suspicion of sepsis had prompted an assessment of lactate, but who had had a normal outcome and thus had a low likelihood of infection (assessed by birth outcomes and proxy laboratory markers).

## 2 METHODS

### 2.1 Population selection

Electronic patient records were searched to identify pregnant women who gave birth at Oxford University Hospitals between 6 March 2015 and 20 February 2021. Using routinely collected clinical data, we then refined this in a stepwise approach to include only women with an objectively low risk of having had intra‐ or peripartum infection. First, the search was limited to women who had lactate measured on the day of delivery, or during the next 24 hours. Lactate is measured in the peripartum period for several reasons, including to investigate anaemia, electrolyte disturbance, and suspected sepsis. Therefore, we then refined the cohort to include women with a low likelihood of infection, based on their outcomes. Inclusion criteria were adult women who delivered live, healthy, singleton babies at term gestations. Exclusion criteria were maternal age <18 years, twin pregnancies and higher multiples, intrauterine, intrapartum or neonatal death, any admission for neonatal care (including short admissions for neonatal antibiotics), delivery before 37 completed weeks of gestation, and missing data on lactate.

Data on lactate, laboratory haemoglobin, WBC and CRP were extracted for eligible participants, and in the event of repeated measurements during the study period, only the first was included in each case. Women were excluded who had WBC >23 × 10^9^/l (or missing data), as results above this level are unlikely to be seen in healthy women despite the physiological leucocytosis in labour.^15^ Women with severe anaemia (<50 g/l) or polycythaemia (>160 g/l) were excluded, due to the increased likelihood of significant concurrent disease. Women with common pregnancy complications were identified as those with hypertensive disorders of pregnancy (ICD‐10 O10‐16), and pre‐existing or gestational diabetes (O24). Finally, we limited the analysis to include only results with <10% fetal haemoglobin, as this effectively excludes any samples that might have been erroneously labelled as maternal blood (e.g. umbilical cord or fetal blood samples).

Lactate was determined on whole blood using the ABL90 Flex analyser (Radiometer UK Ltd) as a point of care instrument on the delivery suite. Haemoglobin and WBC were measured as part of a full blood count using the Sysmex XN analyser (Sysmex UK Ltd). Plasma CRP was analysed using an immunoturbidimetric assay on an Abbott Architect c16000 instrument (Abbott Laboratories Ltd).

### 2.2 Statistical methods

Before undertaking the analysis, the distribution of lactate data was scrutinised in a final step to identify and exclude outliers. As is widely practised in studies of pregnancy‐specific reference intervals, lactate results were transformed to approximate the normal distribution using a Box‐Cox logarithmic transformation, and high outliers were defined as values >1.5 times the interquartile range above the mean (Tukey method). Low lactate outliers were not considered to be clinically relevant.

We summarised the distribution of lactate data and investigated the proportion of women with results >2 and >4 mmol/l. Univariate linear regression was used to investigate associations between lactate and predetermined participant characteristics (maternal age, hypertension and diabetes), and other biomarkers associated with haemoconcentration (haemoglobin concentration and body mass index [BMI]). Subgroup analyses were performed to investigate differences in lactate according to the day of sampling (day 0 or 1) and mode of birth (spontaneous or operative vaginal delivery, elective, or emergency caesarean section).

Women with missing data for secondary characteristics were included. All analyses were performed using native packages in STATA (version 17.0 for Windows, StataCorp LLC). Summary statistics were presented as the mean ± standard deviation for normally distributed variables, or the median and interquartile range (IQR) otherwise. Group differences were investigated with Kruskal–Wallis tests, and statistical significance was assumed where *p* < 0.05. The population size was determined by the total number of women with available data during the study period, and we performed a post‐hoc calculation to investigate the power with which this study was able to investigate the observed group‐specific differences in lactate (the difference between two means, at the 5% confidence level).

### 2.3 Patient involvement

Patients and the public were not involved in the planning or undertaking of this study.

## 3 RESULTS

In total, 37 924 women delivered live, singleton babies in Oxford over a 6‐year period. Of these, 2402 (6.3%) had lactate measured within 24 hours of delivery, and 1318 women met all the inclusion criteria. In accordance with international guidelines,^21^ a small proportion of women with very high lactate were excluded (>5.4 mmol/l, *n* = 39, 3.0%), as these results are likely to represent an underlying pathology. In total, 1279 participants were included in the final analysis. The frequencies and reasons for exclusions are presented in Figure S1, and maternal and fetal characteristics of the included participants are presented in Table 1.

**TABLE 1:** Participant characteristics

Characteristic	Summary data	Missing data (*n*, %)
Number of women	1279	
Maternal age, years	30.7 ± 5.5	
Body mass index, kg/m^2^	26.2 ± 6.0	20 (1.6%)
Ethnicity		
White	968 (75.7%)	39 (3.1%)
Mixed	24 (1.9%)	
Asian	173 (13.5%)	
Black	37 (2.9%)	
Other	38 (3.0%)	
Gestational age, weeks	40.2 ± 1.3	
Neonatal birthweight, kg	3.6 ± 0.5	
Mode of birth		
Spontaneous vaginal	389 (30.4)	
Operative vaginal	397 (31.0)	
Elective Caesarean	96 (7.5)	
Emergency Caesarean	397 (31.0)	
Neonatal sex		
Male	690 (53.9%)	1 (0.1%)
Female	588 (46.0%)	
Total WBC, x 10^9^/L	15.8 ± 3.8	
Haemoglobin, g/l	109 ± 16	
Haematocrit, proportion	0.32 ± 0.05	

**FIGURE 1:**
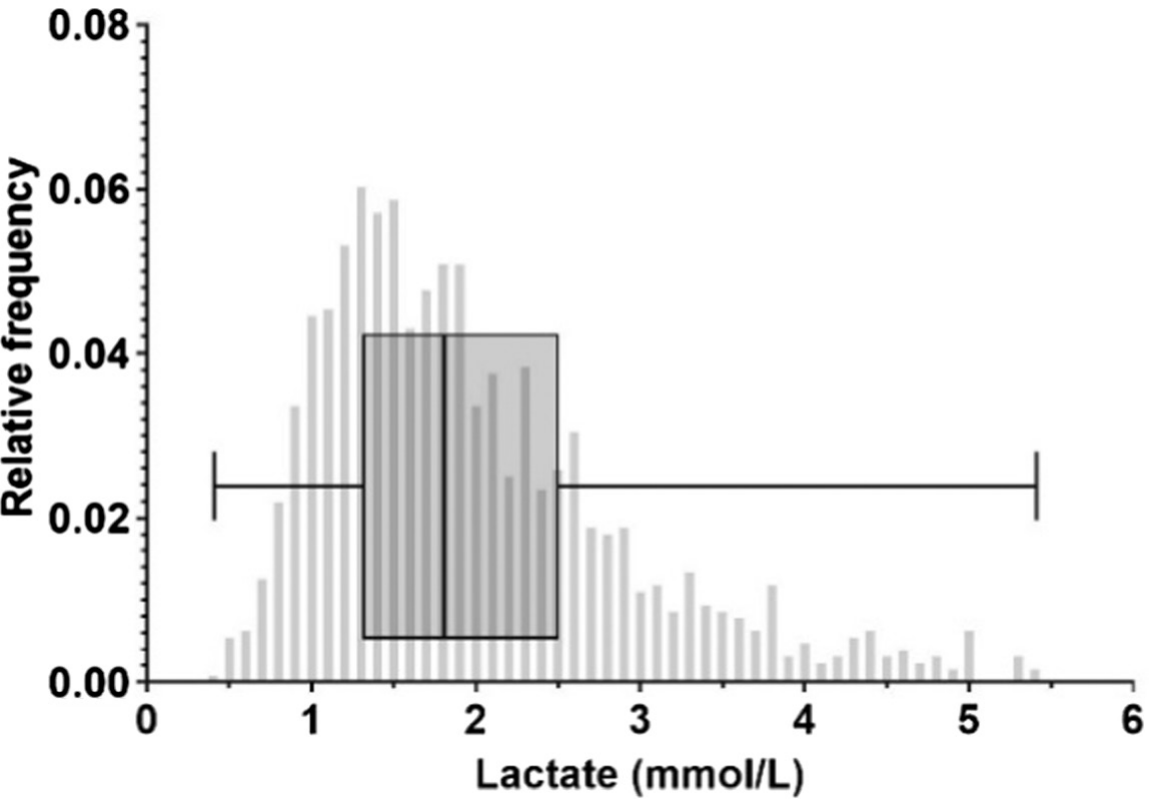
Lactate during labour and delivery. Histogram displaying the distribution of 1279 lactate measurements, with a box‐and‐whisker plot summarising the 25th, 50th and 75th centiles, and minimum‐maximum range.

In the peripartum period, the median lactate concentration was 1.8 mmol/l (IQR 1.3–2.5) and ranged between 0.4 and 5.4 mmol/l (see Figure 1). In total, 522 of these women (40.8%) had a lactate ≥2 mmol/l, and 60 (4.7%) were ≥4 mmol/l.

**FIGURE 2:**
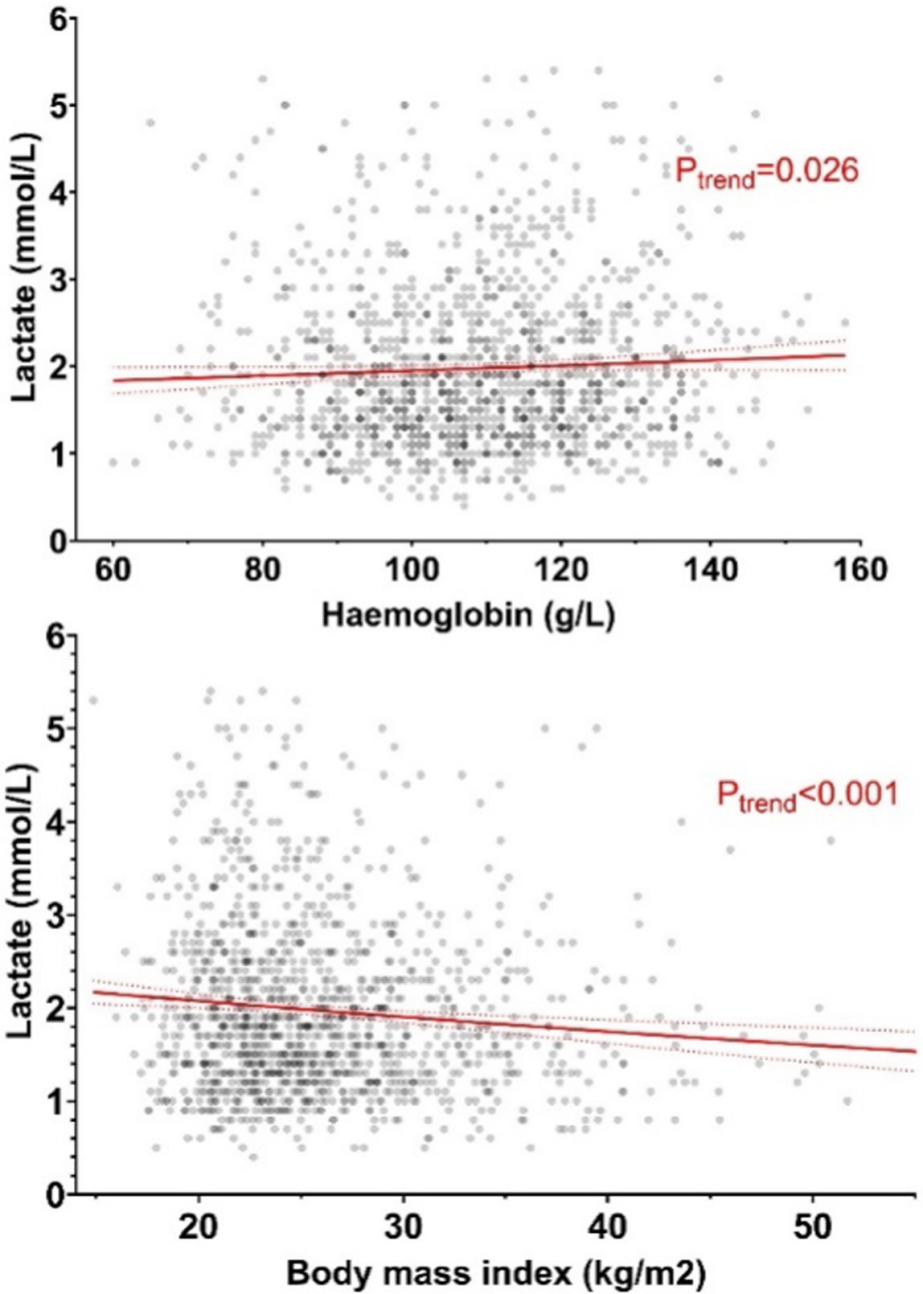
Lactate and haemoconcentration. Scatter charts overlaid with univariate linear estimates between lactate, haemoglobin and body mass index. Caption: Red lines denote linear estimates with 95% confidence intervals around the mean.

Lactate was log‐linearly associated with the haemoglobin concentration (lnRR 0.02 mmol/l per 10% increase, 95% CI 0.00 to 0.03, *p* = 0.026), and inversely associated with BMI, as a proxy marker of plasma volume (lnRR −0.08 mmol/l per additional 10 kg/m^2^, 95% CI −0.12 to −0.04, *p* < 0.001), as shown in Figure 2. Lactate was associated with increased maternal age (lnRR 0.07 per additional 10 years, 95% CI 0.03–0.12, *p* = 0.002), but not with having any hypertensive disorder (lnRR −0.05, 95% CI −0.12 to +0.02, *p* = 0.181) or diabetes (lnRR −0.04, 95% CI −0.13 to +0.05, *p* = 0.334).

### 3.1 Subgroup analysis and CRP

**FIGURE 3:**
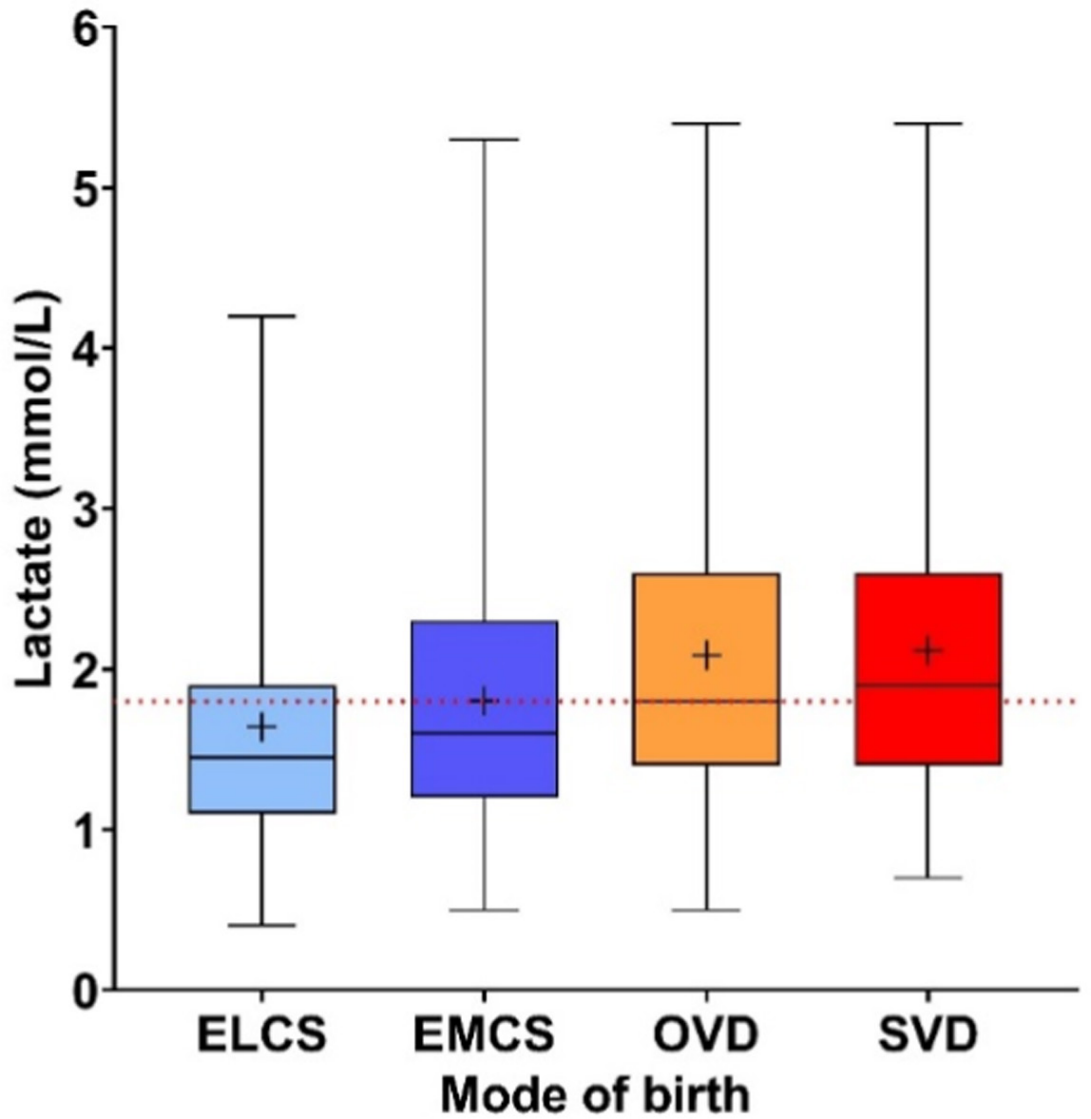
Lactate and mode of birth. Box‐and‐whisker plots for lactate measurements according to mode of birth: elective caesarean (*n* = 96), emergency caesarean (*n* = 397), operative vaginal delivery (*n* = 397) or spontaneous vaginal delivery (*n* = 389). Caption: Red dotted line denotes the median for all women, and crosses (+) represent the group‐specific averages. Boxes on the distribution plots represent the 25th, 50th and 75th centiles, and whiskers demarcate the minimum–maximum values.

Lactate was significantly higher on the day of delivery than on the following day (median 1.9 vs. 1.4 mmol/l, *p* < 0.001, see Figure S2). Overall, lactate varied significantly according to the mode of delivery in at least one group (*p* < 0.001, see Figure 3): the median lactate was higher in women who had vaginal deliveries (1.9 mmol/l, IQR 1.4–2.6) than in women who delivered by caesarean section (1.6 mmol/l, IQR 1.2–2.2, *p*_diff_ < 0.001). There was no significant difference between spontaneous or assisted vaginal delivery (1.9 vs. 1.8 mmol/l, *p*_diff_ = 0.586), but our study was not powered to investigate this (29% power). Similarly, the difference between elective and emergency caesarean was not statistically significant (1.5 vs. 1.6 mmol/l, *p*_diff_ = 0.095, 14% power).

Of the 709 women (55.4%) who also had CRP measured, the median was 42.8 mg/l (IQR 17.6–91.1). This was significantly higher on day 1 than day 0 (121.2 vs. 33.3 mg/l, *p*_diff_ < 0.001). Overall, CRP was higher in women who gave birth by caesarean section than those who had vaginal deliveries (71.8 vs. 33.4 mg/l, *p* < 0.001).

## 4 DISCUSSION

### 4.1 Main findings

Current UK guidelines on perinatal sepsis recommend that a maternal lactate >4 mmol/l indicates life‐threatening tissue hypoperfusion and end‐organ damage. This should prompt urgent treatment, including intravenous antibiotics and fluid resuscitation.^12^, ^13^ Our study found that very few pregnant women had a lactate >4 mmol/l at any point (specificity 95.3%). However, NICE guidance recommends a lower threshold of 2 mmol/l to identify patients for whom antimicrobial therapy should be considered. Based on our findings, this lower threshold is only 59.2% specific for infection during labour or after childbirth. In other words, almost half of the women in this study (whose outcomes were not consistent with perinatal infection) had a lactate >2 mmol/l, and the diagnostic uncertainty that this introduces may have detrimental effects on decisions about antibiotic prescriptions, surgical intervention (as a result of disease misclassification) and the use of broad‐spectrum antibiotics for newborn babies.

### 4.2 Interpretation

It has been reported that lactate concentrations increase during the second stage of labour in otherwise healthy women, particularly with active pushing.^22^, ^23^ While fetal lactate concentrations also rise in the second stage of labour, the elevated levels in maternal blood are reportedly independent and predominantly maternally derived, driven by intermittent myometrial hypoxia.^24^ This is broadly consistent with studies in other specialties: lactate is well‐described as a product of physical exertion, particularly in short‐term, high‐intensity exercise, and also as a substrate for skeletal muscle.^25^ Indeed, lactate turnover is of particular interest in sports medicine, as an understanding of the dynamic changes seen in lactate may be used for performance management.^26^ In our study, women in labour who pushed for any duration (those who had vaginal deliveries) had higher lactate values than those who did not (elective caesarean sections). Unsurprisingly, lactate in women who had emergency caesarean sections (many of whom will have been in labour and/or pushed, but for a shorter duration) was inbetween these groups. This is consistent with small studies that have reported a dose–response association between lactate and the duration of the first and second stages of labour.^22^ Furthermore, we found that temporal proximity to pushing was also associated with lactate, illustrated by higher levels on the day of delivery than on the following day.

Further to the investigation of (bacterial) sepsis, lactate has an important role in the assessment of systemic wellbeing and outcome prediction in other diseases in both pregnant and non‐pregnant women. For example, features of severe malaria include haemolytic anaemia, dehydration, haemoconcentration and lactic acidosis (note the differentiation between serum lactate and lactate dehydrogenase, a potential diagnostic marker for malaria).^27^, ^28^ We have demonstrated linear associations between lactate and maternal haemoconcentration in non‐infected women in labour. We used routinely collected clinical data to illustrate the underlying mechanism: pregnant women with a higher haemoglobin concentration had higher lactate levels, but lactate was lower in association with a raised BMI (driven by the relatively greater plasma expansion and haemodilution in obese women).

Lactate is one of several non‐specific ‘infection’ markers used in current clinical practice, the most common of which is CRP. In this study, CRP in the peripartum period was raised substantially above the antenatal pregnancy‐specific reference interval,^14^ with a further marked elevation after caesarean birth. Unlike lactate, which was highest on the day of delivery, CRP continued to rise, with ‘late peak’ on the first postnatal day. A reference study by Joyce et al.^16^ reported widely ranging CRP values in the immediate postnatal period, with more than a fifth (21.1%) of healthy women having a CRP >100 mg/l on the day after a caesarean section. There is a substantial and growing body of evidence to support that CRP and WBC (both notoriously non‐specific markers) have limited value after the onset of labour, and there is an unmet need for reliable alternatives.^15^ Procalcitonin is a highly specific marker for bacterial infection, which is widely used to support decisions about antibiotics, and as a marker of severity and prognosis in pregnancy‐associated sepsis.^29^ Importantly, it can be interpreted using the same cut‐offs in both pregnant and non‐pregnant women, at all stages of pregnancy and the puerperium.^10^, ^16^ Unfortunately, procalcitonin is not yet used in routine obstetric practice, although there may be a role for procalcitonin‐driven decision making in pregnancy, in line with established pathways in other specialities.^9^

### 4.3 Strengths and weaknesses

We investigated the distribution of lactate in a large, well‐defined cohort of pregnant women. The study population is comparable with UK demographics with respect to maternal age,^30^ ethnicity^31^ and BMI.^32^ The interpretation of lactate (and other diagnostic tests) poses a real challenge in pregnancy, and the aim of this study is to clarify some of the inconsistencies in the published literature, to support safe, evidence‐based clinical decision making.

In this pragmatic observational study, lactate was measured when indicated as part of routine clinical care, and pregnant women with good outcomes were retrospectively selected using extensive data on maternal characteristics and proxy markers. On one hand, this is a limitation, as it is not a prospective study of lactate in women selected according to a definition of ‘health’, and lactate is not routinely measured in uncomplicated labour. However, this is also a strength, as the results of this study are directly relevant to the population of pregnant women for whom lactate is usually measured. It is challenging to establish the presence or absence of infection without data on microbiological growth, antibiotic prescriptions and hospital readmissions. However, there is precedent for using indirect retrospective data to investigate pregnancy‐specific reference intervals,^15^ and this approach allows a deeper investigation into the associations and underlying physiological mechanisms, which has often not been possible in smaller studies.

Lactate continues to have an important role in the management of sepsis in pregnant women. It is usually primarily intended as a marker of severity, but there is a paucity of evidence for using it to inform decisions about antibiotics. Importantly, raised lactate levels in labour, even when <4 mmol/l, may still represent sepsis with tissue hypoperfusion. It is thus essential to consider lactate results in the context of the wider clinical picture, remaining cautious that elevation above the usual (non‐pregnant or antenatal) levels may be physiological or pathological.

## 5 CONCLUSION

This study describes the normative distribution of lactate levels around the time of delivery. Lactate in labour and the puerperium is commonly elevated above the levels expected in healthy pregnant or non‐pregnant women (>2 mmol/l), but it is rarely >4 mmol/l.

## AUTHOR CONTRIBUTIONs

SD: conception, methodology, data analysis, manuscript writing and revision. JO'S: data analysis, manuscript writing and revision. BS: data procurement, manuscript writing and revision. TJ: conception, manuscript writing and revision. MV: conception, manuscript writing and revision, supervision. All authors accept responsibility for the paper as published.

## FUNDING INFORMATION

None.

## CONFLICT OF INTERESTS

None declared. Completed disclosure of interest forms are available to view online as supporting information.

## ETHICAL APPROVAL

Ethics approval was granted by the Health Research Authority Research Ethics Committee, Oxford South Central C (Ref: 08/H0606/139), and informed consent was not required.

## Supporting information


Figure S1
Figure S2.

Appendix S1

Appendix S2

Appendix S3

Appendix S4

Appendix S5

## Data Availability

The data that support the findings of this study are available on request from the corresponding author. The data are not publicly available due to privacy or ethical restrictions.
