# Supplementary material for: How should we interpret lactate in labour? A reference study
Source: BJOG. 2022 Aug 8;129(13):2150–6. doi: 10.1111/1471-0528.17264 (PMC9804290; doi:10.1111/1471-0528.17264)
Supplement: Supplementary file 1 — Figure S1 Figure S2. [file BJO-129-2150-s001.docx]

# Supporting Information

# Figure S1: Flow diagram for selecting participants

All singleton deliveries n=37,924

Lactate not measured n=35,522

Women with available lactate data

n=2,402

**Exclusions n=1,084**

WBC not measured n=204

HbF data unavailable n=180

Age<18 years n=22

SCBU admission n=333

Preterm delivery n=81

Leucocytosis n=216

Extreme anaemia or polycythaemia n=48

Potentially eligible

n=1,318

**Outliers n=39**

High n=39

Women included

n=1,279

# Figure S2: Subgroup analysis. Box-and-whisker plots summarising the distribution of lactate measured on the day of delivery (n=1028) and the following 24 hours (n=251).


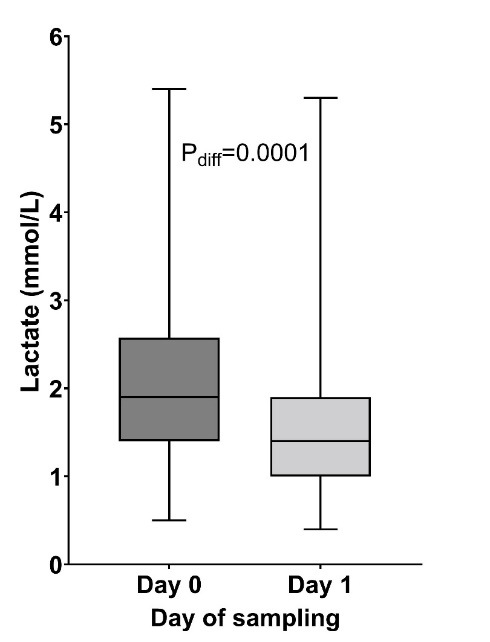


P-value derived from a Kruskall-Wallis non-parametric (Chi^2^) test for group differences.
